# Post-Topotecan Mixed Response and ‘Redifferentiation-akin’ Phenomenon on Dual Tracer PET-CT in Multiple Treatment-Resistant Metastatic Neuroendocrine Neoplasm

**DOI:** 10.1055/s-0043-1760761

**Published:** 2023-04-28

**Authors:** Sarvesh Loharkar, Sandip Basu

**Affiliations:** 1 Radiation Medicine Centre, Bhabha Atomic Research Centre, Tata Memorial Hospital Annexe, Parel, Mumbai, Maharashtra, India; 2 Homi Bhabha National Institute, Mumbai, Maharashtra, India

**Keywords:** neuroendocrine neoplasm, topotecan, ^68^ Ga-DOTATATE, PET-CT, peptide receptor radionuclide therapy

## Abstract

A 50-year-old female patient of heavily pre-treated (chemotherapy and multiple treatment-resistant) and progressive intermediate-grade metastatic neuroendocrine neoplasm is presented, wherein the lesions showed mixed response following topotecan treatment and multiple hepatic metastasis showed increase in the SSTR expression and decrease in FDG concentration on dual-tracer PET/CT ( ^68^ Ga-DOTATATE and ^18^ F-FDG PET/CT). Such observation allowed ^177^ Lu-DOTATATE PRRT to be considered for an advanced, symptomatic, and multiple treatment-resistant patient with limited palliative treatment options left.

## Introduction

The heterogeneity of the neuroendocrine neoplasms (NENs) varies between the complete spectrum of well-differentiated to poorly differentiated tumors/carcinomas. The recently adopted 2021 WHO classification gave two separate categories of high-grade (grade 3) NENs as well-differentiated NEN and poorly differentiated NEN/neuroendocrine carcinoma (NEC). This new concept replaced the previous definition of grade-3 NEN based purely on the K _i_ -67 level and/or mitotic index as NEC. 1 The behavior, prognosis and selection of the correct treatment option for these tumors change significantly with their differentiation, with NECs considered the most aggressive subsets of NENs. 2 Peptide receptor radionuclide therapy (PRRT) with ^177^ Lu-DOTATATE has evolved as an important treatment option in well-differentiated and intermediate-grade NETs that show adequate somatostatin receptor (SSTR) expression on PET-CT imaging. However, some intermediate-grade NENs showing minimal/low-grade SSTR positivity makes them unsuitable for PRRT. It has been observed and reported that certain forms of chemotherapy may induce a (i) radiosensitizing effect in tumor cells, which eventually increases the tumor response after PRRT, and also (ii) may enhance tracer uptake on SSTR-based PET-CT. 3 4 Commonly used chemotherapeutic agents where such effects are described include capecitabine, temozolomide, and 5-fluorouracil. The first effect of radiosensitization has been related to increased DNA damage and inhibition of DNA repair, inhibition of cell proliferation, tumor cell re-oxygenation, apoptosis, or synchronization of the cell cycle. The latter, on the other hand, is vastly unexplained at present. Following such ‘redifferentiation-akin’ effect, these patients may be eventually targeted with PRRT to achieve maximum therapeutic benefit. 3 4 We herein present an aggressive metastatic rectal NEN with low SSTR uptake initially and resistant to multiple chemotherapy regimens and liver-directed therapies, showed mixed response and ‘re-differentiation-akin’ phenomenon following topotecan.

## Case Report

A 50-year-old female patient, initially diagnosed with poorly differentiated rectal NEC with large soft tissue nodal mass in the left pelvic wall, received standard chemotherapy regimen of six cycles of etoposide and carboplatin previously. In view of non-response, she was evaluated for PRRT. ^99m^ Tc-HYNIC-TOC scan revealed low-moderate grade uptake in the left-sided pelvic mass, and histopathology and IHC review suggested a Mib-1 labeling index of 10%. Considered for chemo-PRRT, she received a total of 10 cycles of oral chemotherapy with capecitabine-temozolomide (CAPTEM) and 4 cycles PRRT (cumulative activity of ^177^ Lu-DOTATATE received was 705 mCi/26.08 Gbq), with no significant change in size and intensity of SSTR expression in rectal primary, left external iliac nodal mass, and left pelvic nodes on ^68^ Ga-DOTATATE-PET/CT. Surgical opinion was declined in view of inability to achieve R0 resection. Following a stable disease for the next 1.5 years, she developed a solitary liver lesion, which on USG-guided biopsy revealed it to be NET-grade II (Mib-1 index: 9–10%). The patient underwent lipiodol-TACE of the right lobe liver lesion (segment-V) using 50 mg doxorubicin with 10 mL of lipiodol. Follow-up liver MRI showed post-TACE changes in the lesion but also revealed a few other tiny lesions in the liver, for which multiple TACE and CT-guided RFAs were undertaken over next 2 years along with octreotide LAR, but recurrence developed in a very short period. The pelvic lesion was stable during this time while persistent progression of the liver lesions with an increase in FDG concentration in most lesions but very few lesions were adequately SSTR expressing on ^68^ Ga-DOTATATE PET/CT. The patient experienced significant weight loss, constipation, and occasional abdominal pain. Also noted was obstructive uropathy due to left-sided iliac mass with left-sided kidney showing a serial reduction in cortical function. (The therapies received are summarized in Table 1 .)

**Table 1:** Summary of therapies received and their timeline

Sr. No.	Therapy details	Period
1.	Diagnosed with metastatic rectal NEC after 1 month of rectal symptoms	May 2014
2.	Received chemotherapy regimen of 6# of etoposide and carboplatin (disease stabilization)	Until Dec 2014
3.	^99m^ Tc-HYNIC TOC scan and histopathological re-evaluation	In Feb 2015
4.	Received chemo–PRRT as per SSTR uptake on scan and 10%MiB index on histopathology 4# of PRRT ( ^177^ Lu-DOTATATE) 11# of CAP-TEM oral chemotherapy (variable intervals and dosage considering toxicities) Follow by observation only for 1.5 years (disease stabilization)	Until April 2016Until Nov 2016 Until May 2018
5.	Diagnosed with non SSTR expressing liver lesion on ^68^ Ga-DOTATATE PET/CT, Grade II NET as per USG guided Biopsy	June 2018
6.	Lipiodol-TACE for liver lesions	Sept 2018, Nov 2018, Jan 2019, and Oct 2019
7.	CT guided RFA	April 2019, June 2019, and Oct 2019
8.	Observation and follow-up with liver MRI and dual tracer PET/CT; received octreotide LAR (Delayed follow-up due to the COVID-19 pandemic)	Until Dec 2020
9.	(Progression and symptomatic for left iliac nodal mass)-palliative external radiation (8Gy) to iliac nodal mass	Jan 2021
10.	(Delayed follow-up due to COVID-19 pandemic) trial of topotecan	Sept 2021 to Dec 2021
11.	Response evaluation dual tracer PET/CT (post Topotecan)	Feb 2022
12.	Salvage PRRT	March 2022

**Fig. 1:**
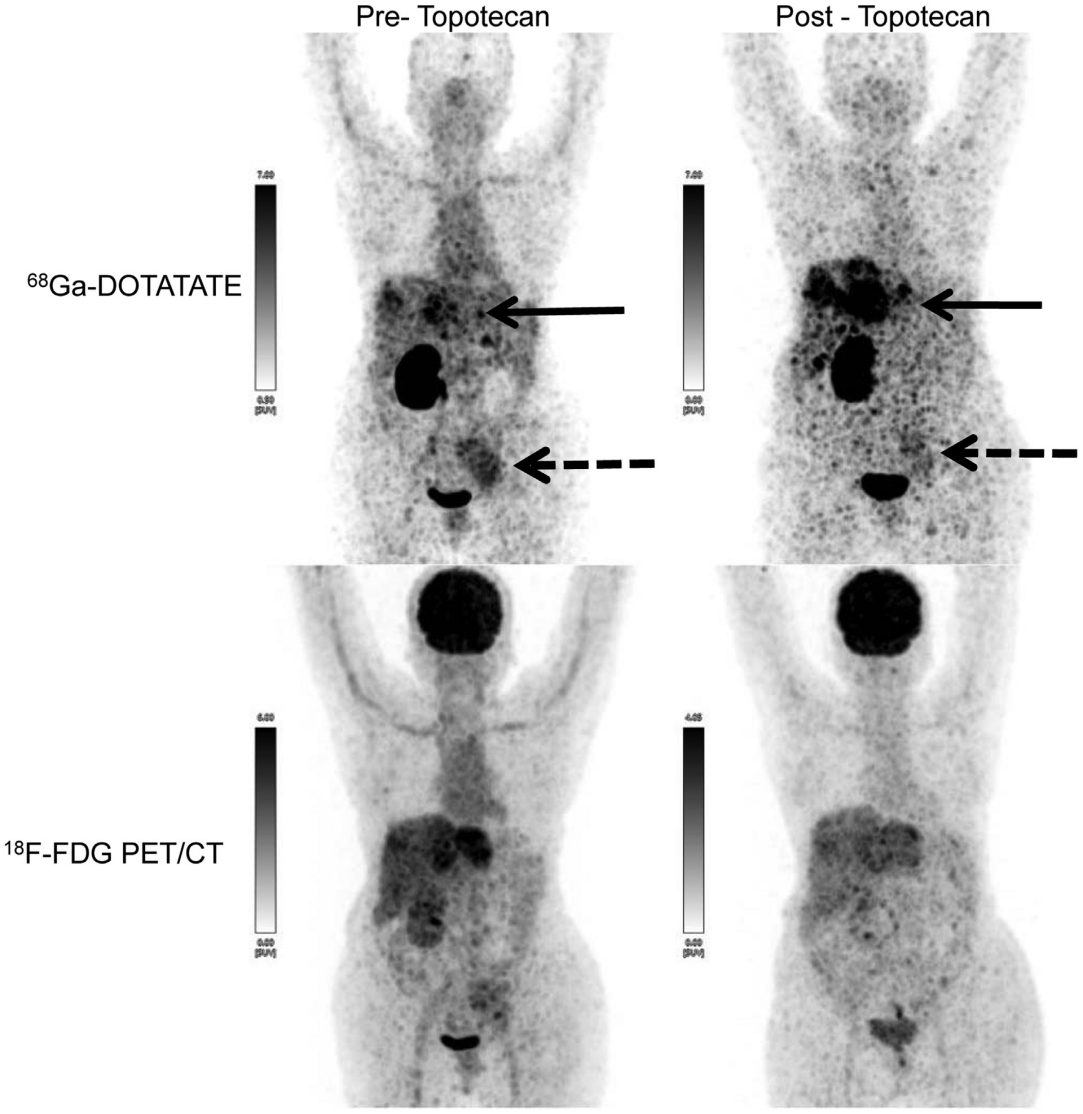
^68^ Ga-DOTATATE and ^18^ F-FDG PET/CT MIP images pre- and post-topotecan chemotherapy, showing increase in the SSTR expression in liver lesions ( *solid arrow* ), while decrease in left-sided iliac nodal lesion ( *dashed arrow* ), and decrease in FDG uptake in liver lesions.

**Fig. 2:**
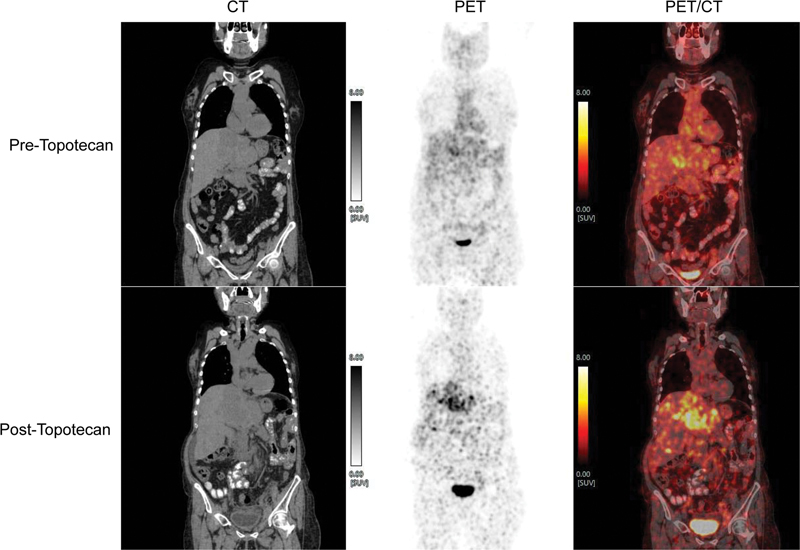
^68^ Ga-DOTATATE-PET/CT pre-and post-topotecan therapy (coronal CT, PET, and fused PET/CT images) showing increase in the SSTR expression of the liver lesions. (SUVmax for liver segment II lesion post-topotecan therapy is now 9.7 vs. pre-topotecan therapy was 6.2.)

**Fig. 3:**
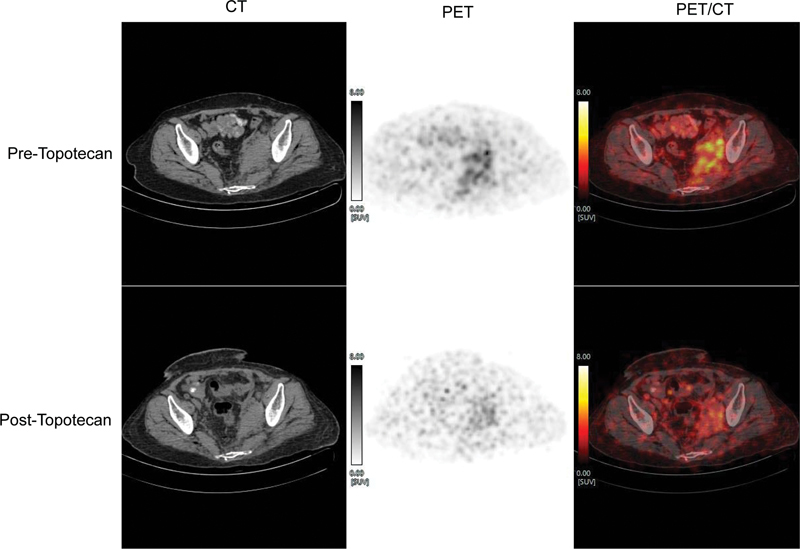
^68^ Ga-DOTATATE-PET/CT pre-and post-topotecan therapy (axial CT, PET and fused PET/CT images) showing a decrease in size and SSTR expression of left-sided iliac soft tissue nodal lesion. (SUVmax for left internal iliac nodal mass post-topotecan therapy is now 6.3 versus pre-topotecan therapy was 17.7.)

In view of being symptomatic, rechallenge-treatment was considered with systemic chemotherapy, while PRRT was differed considering very low SSTR expression ( Fig. 1 ). She received palliative external radiation (8 Gy) to iliac nodal mass due to symptoms of lower limb edema and walking difficulties. Due to symptomatic progression and non-responder to multiple previous chemotherapies, she was trialed with Topotecan. She received three cycles of Topotecan in 3 months. This was planned as two cycles every 28 days but spaced considering grade II hyponatremia, and hyperkalemia with hematological toxicity. Considering transient grade III hematotoxicity her further therapy was stopped. She recovered with the blood counts soon. A response evaluation dual-tracer PET/CT showed a mixed response (pre- and post-topotecan therapy scans Figs. 1 , 2 , 3 ) with marginal increase in the size of multiple liver lesions (with a few new areas of hypodensities/necrotic regions and a few central calcific changes are noted) and also in SSTR expression, and decrease in FDG concentration. Left-sided internal iliac nodal mass ( Fig. 3 ) showed reduction in size, SSTR expression, and FDG concentration (uptake-wise SSTR > FDG).

**Fig. 4:**
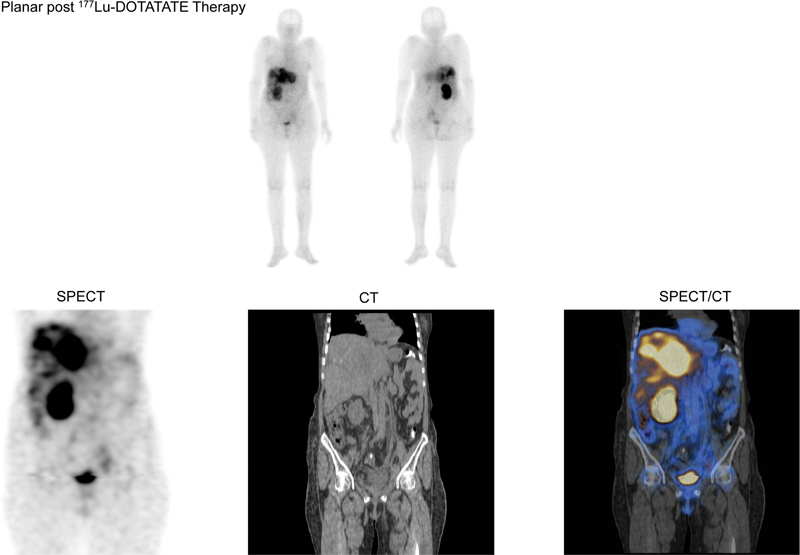
Post- ^177^ Lu-DOTATATE therapy planar gamma camera and regional (abdomen and pelvis) SPECT/CT images showing tracer concentration in liver lesions.

Considering these findings, the increase in the SSTR expression and previous experience with multiple therapies she was planned for salvage PRRT in reduced dose considering the single functioning renal status. She received 157 mCi (5.8 GBq) of ^177^ Lu-DOTATATE IV and tolerated the therapy well, the post-therapy scan showed adequate liver uptake ( Fig. 4 ). She experienced symptomatic response and both blood and renal parameters in stable range as last evaluated post-1 month of therapy.

## Discussion

Topotecan is an inhibitor of topoisomerase I and acts by forming a stable covalent complex with the DNA/topoisomerase I aggregate, leading to breaks in the DNA strand, apoptosis, and cell death. It has been tried successfully in multiple tumors, especially in chemotherapy-resistant and heavily pre-treated patients with non-small cell lung cancer, ovarian, colon, and other solid tumors. Multiple groups have tried it in poorly differentiated and advanced heavily pre-treated NENs. 5 6 Similar situation was encountered in our case wherein the disease demonstrated progression following multiple therapies, following which topotecan was considered. Interestingly, the case showed a mixed anatomical response (pelvic lesions showed partial anatomical response while the liver lesions showed increased size and number).

There is paucity of literature regarding response evaluation post-topotecan therapy in heavily pre-treated NEN patients and such chemotherapy-induced increased SSTR expression on SSTR-based PET/CT. To our knowledge, post-topotecan increase in SSTR expression in lesions on ^68^ Ga-DOTATATE-PET/CT is not elucidated in the literature and this would be first such literature-report in advanced, treatment-resistant NENs. In the previously reported short case series, similar such observation was described in the context of CAPTEM and everolimus. 3 4

## Conclusion

In conclusion, heavily pre-treated and treatment resistant NENs may show a response to topotecan and could be better evaluated with dual tracer PET ( ^68^ Ga-DOTATATE and ^18^ F-FDGPET/CT), wherein the observation of increased uptake and SSTR expression on tumor cells could open up suitable therapeutic avenues such as PRRT in such scenarios.
